# Finite element analysis and clinical evaluation of cross locking external fixator configuration for distal third tibia fracture

**DOI:** 10.1038/s41598-025-97090-4

**Published:** 2025-04-17

**Authors:** Bing Wui Ng, Abdul Hadi Abdul Wahab, Abdul Muttalib Abdul Wahid, Nik Nur Ain Azrin Abdullah, Mohammed Rafiq Abdul Kadir, Muhammad Imam Ammarullah, Muhammad Hanif Ramlee

**Affiliations:** 1 Department of Orthopaedics and Spine, Prince Court Medical Centre, Kuala Lumpur, 50450 Federal Territory of Kuala Lumpur Malaysia; 2 Center for Multimodal Signal Processing, Faculty of Engineering and Technology, Tunku Abdul Rahman University College, Setapak, 53300 Federal Territory of Kuala Lumpur Malaysia; 3 Department Electrical and Electronics Engineering, Faculty of Engineering and Technology, Tunku Abdul Rahman University College, Setapak, 53300 Federal Territory of Kuala Lumpur Malaysia; 4 Department of Orthopaedics, Hospital Segamat, Ministry of Health Malaysia, Segamat, 85000 Johor Malaysia; 5 Bone Biomechanics Laboratory (BBL), Department of Biomedical Engineering and Health Sciences, Faculty of Electrical Engineering, Universiti Teknologi Malaysia, Johor Bahru, 81310 Johor Malaysia; 6 Department of Biomedical Engineering, Faculty of Engineering, Universiti Malaya, Kuala Lumpur, 50603 Federal Territory of Kuala Lumpur Malaysia; 7 Department of Mechanical Engineering, Faculty of Engineering, Universitas Diponegoro, Semarang, 50275 Central Java Indonesia; 8 Undip Biomechanics Engineering & Research Centre (UBM-ERC), Universitas Diponegoro, Semarang, 50275 Central Java Indonesia; 9 Bioinspired Devices and Tissue Engineering (BIOINSPIRA) Research Group, Universiti Teknologi Malaysia, Johor Bahru, 81310 Johor Malaysia

**Keywords:** Cross-locking fixator, Tibia fracture, Finite element, Micromovement, Bone healing, Anatomy, Health care, Engineering, Biomedical engineering

## Abstract

External fixators have been used effectively in damage control orthopaedic and open fractures management of various bones. It is well known that stability of external fixators is greatly influenced by its construct. Various rules have been documented to influence the stiffness and stability of external fixators. In this study, two clinical cases treated with a novel concept of cross self-locking rods external fixation construct were being described, coupled with biomechanical analysis of its stability in comparison with other constructs by using finite element study. These novel self-locking rods configuration proven improve strength by applying the same numbers of rod and pin with the delta frame construct in clinical practice. A validated three-dimensional (3D) model of the bone from a previous study was used and external fixator were designed via computer-aided design (CAD) modelling software, Solidworks. A 1500 N load representing the axial load compression during weight bearing was applied to the tibia with the distal segment of the fracture site secured without any movement. The clinical results showed bone healing process with both cases achieving bone union within the acceptable time. The results of the finite element study shows that the double cross self-locking rods construct had better stability since it showed optimum magnitude in relative micromotion (0.18 mm), lowest stress at the fracture site (189 MPa), displacement of fixator (13.4 mm), and stress at the fixator (687 MPa). In conclusion, double cross self-locking design could provide optimum stability of the external fixator construct by providing better stress distribution at the bone and external fixator, minimize displacement and micromotion at fracture fragments.

## Introduction

The paradigm shift towards soft tissue management in trauma has made external fixation of the bone a commonly employed technique when dealing with open fractures or severe trauma, both in the form of a temporizing device or definitive fixation^1^. The implementation of an external fixator enables wound care, additional reconstructive or plastic procedures to be performed, and permits visualization of fracture site in radiograph, all without loss of reduction at the fracture site^2,3^. The basic principles of the external fixator device are fixation while avoiding the neurovascular structures, stable to fulfil the mechanical demand and to allow access to the area of injury^4^. Rigidity of the construct is an important element of biomechanics to maintain fracture reductions, to allow stability during the range of movement of the limb, and to withstand weight bearing. The factors which could influence the rigidity of the external fixator construct include size of pins, size of rods, distance of rods from the bone, pin-to-pin distance, multiple use of rods and pins, and the material of external fixator^5,6^.

As shown in previous studies, surgeons utilizing external fixators in treating fractures had shown successful bone healing process^7^. Various types of external fixator configurations had been described and used in clinical practice based on factors such as fracture types, patient’s clinical condition, surgical experiences, and the availability of components such as pins and rods^5,8^. These medical devices had become promising treatment options for patients to avoid complications such as wound infections, misalignment, mal-union, and stress shielding effects^9,10^. The unilateral construct is normally used to treat simple fractures^11^. Complex fractures could be stabilized by utilizing multiplanar constructs^12^. In term of biomechanical features, many engineers and researchers have been investigating the properties of external fixator constructs ranging from the overall rigidity to stress distribution, strain or micromotion at the bone, and many other parameters^13,14^. Even though the clinical outcomes are promising, the construct of external fixator can be biomechanically improved by considering factors such as configurations and numbers of component used while at the same time provide a more optimum environment for bone healing and convenience for the patients during the healing process.

In this study, a novel technique to increase the rigidity of the external fixation construct was explored by employing a double cross self-locking rods construct with minimal number of components. The rigidity of this new construct was studied using finite element analysis (FEA) to prove its superior stability. FEA is a computational method which could done repeatedly to evaluate biomechanical properties and prognosticate outcome for various types of injury, surgical technique and types of implant fixation by adjusting the finite element settings of boundary conditions and material properties^15–17^. This method has been utilized in many orthopaedic-related research from previous literature. The models are then converted into FE where simulation with physiological loading was done and the results analyzed to anticipate the outcome of the surgery^13,18^. The FE study was conducted with a combination analysis of two clinical cases to provide further insight on this new external fixator configuration by assessing several parameters such as stress, displacement, and relative micromotion. By having this new qualitative and quantitative data, surgeons will be able to utilize the obtained FEA results to decide on their choices of external fixator construct before a surgery. On the other hand, engineers and researchers will also benefit from this study and plan for further improvement studies.

## Materials and methods

All patients that involved in this study have signed a consent form. The study was conducted according to the guidelines of the Declaration of Helsinki and ethically approved by the National Institutes of Health, Ministry of Health Malaysia.

### Diagnosis and treatment for patient A

**Fig. 1:**
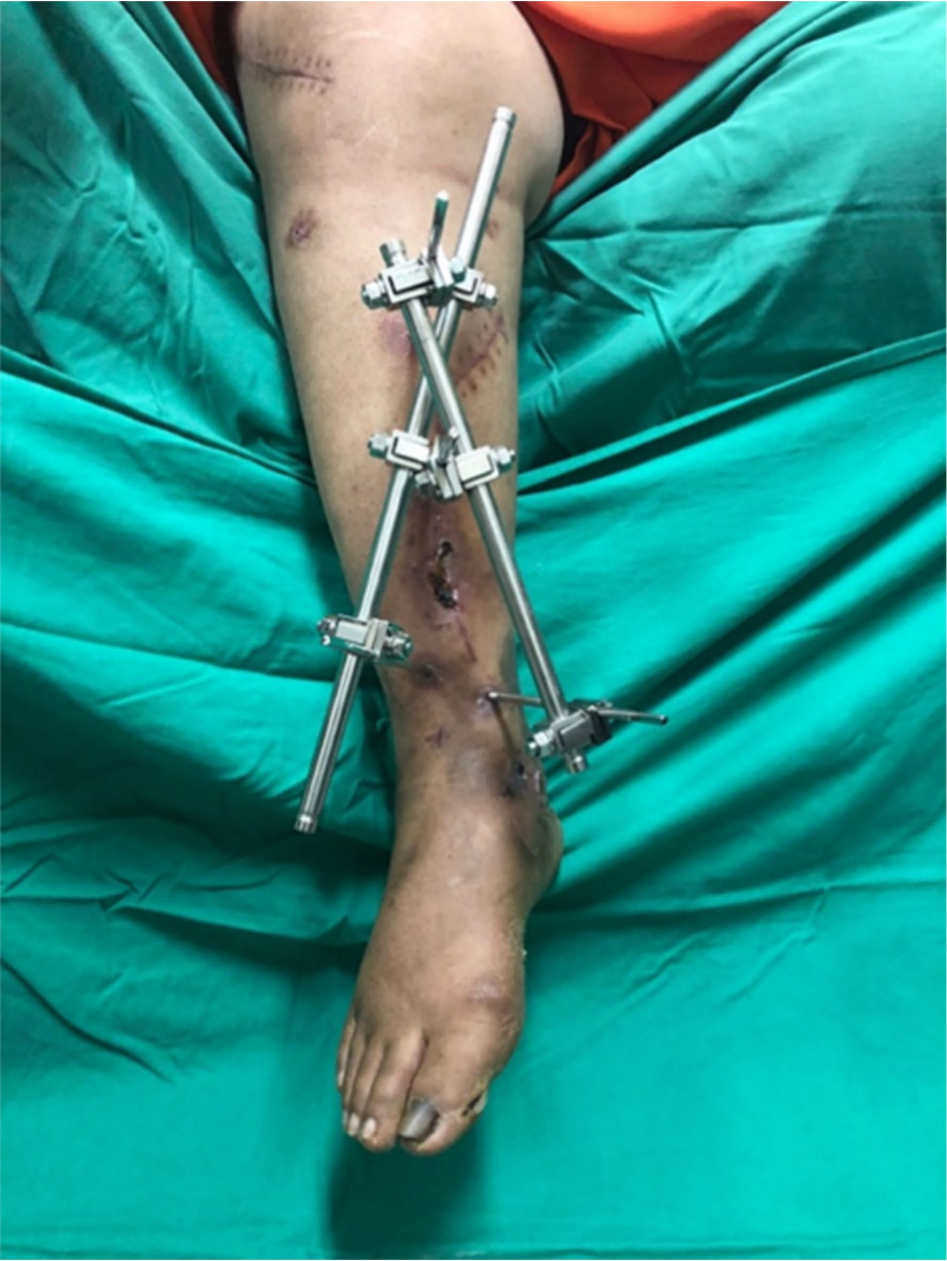
Clinical picture of external fixation construct with rods crossed and locked at both proximal pins.

**Fig. 2:**
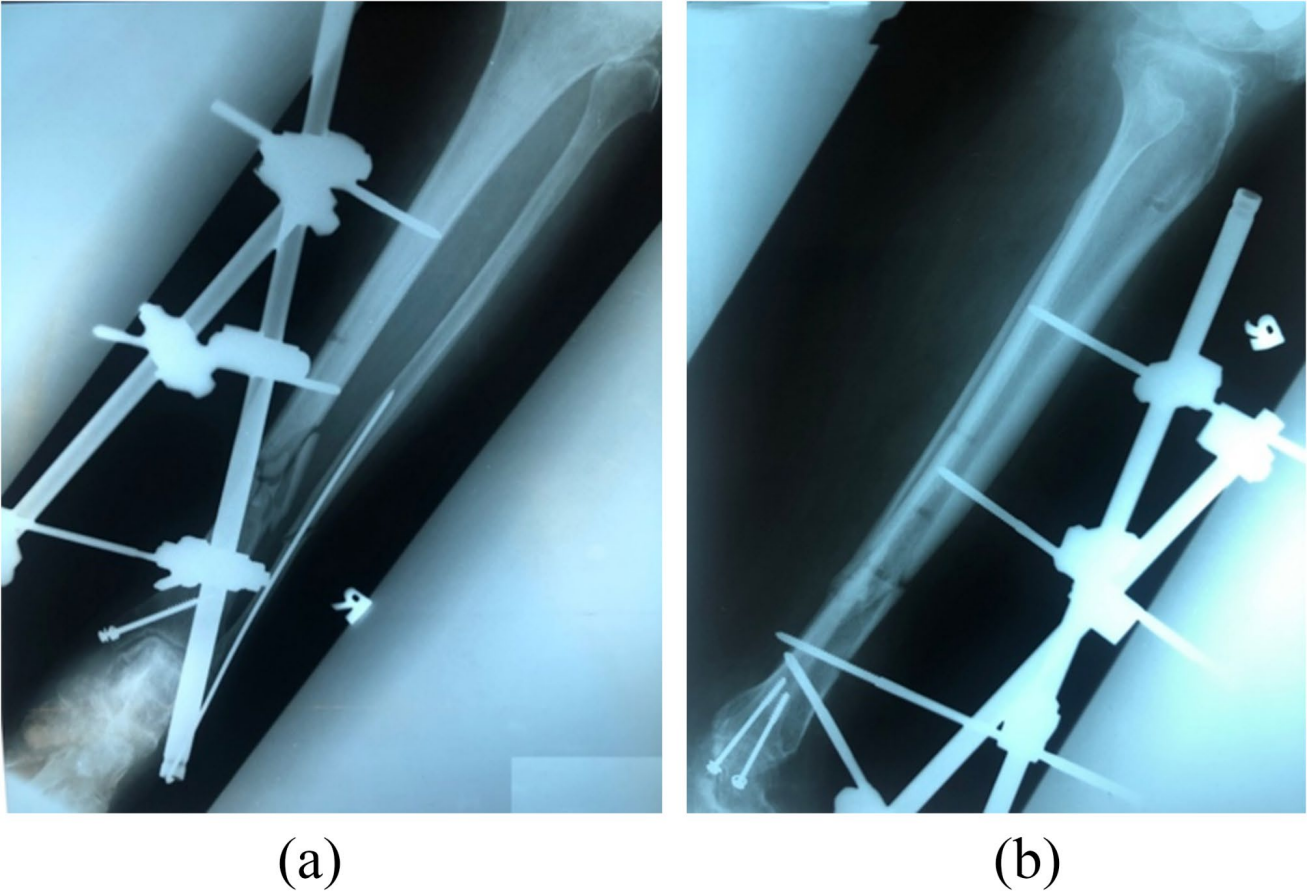
Radiographs of the locking crossed rods done on Patient A.

**Fig. 3:**
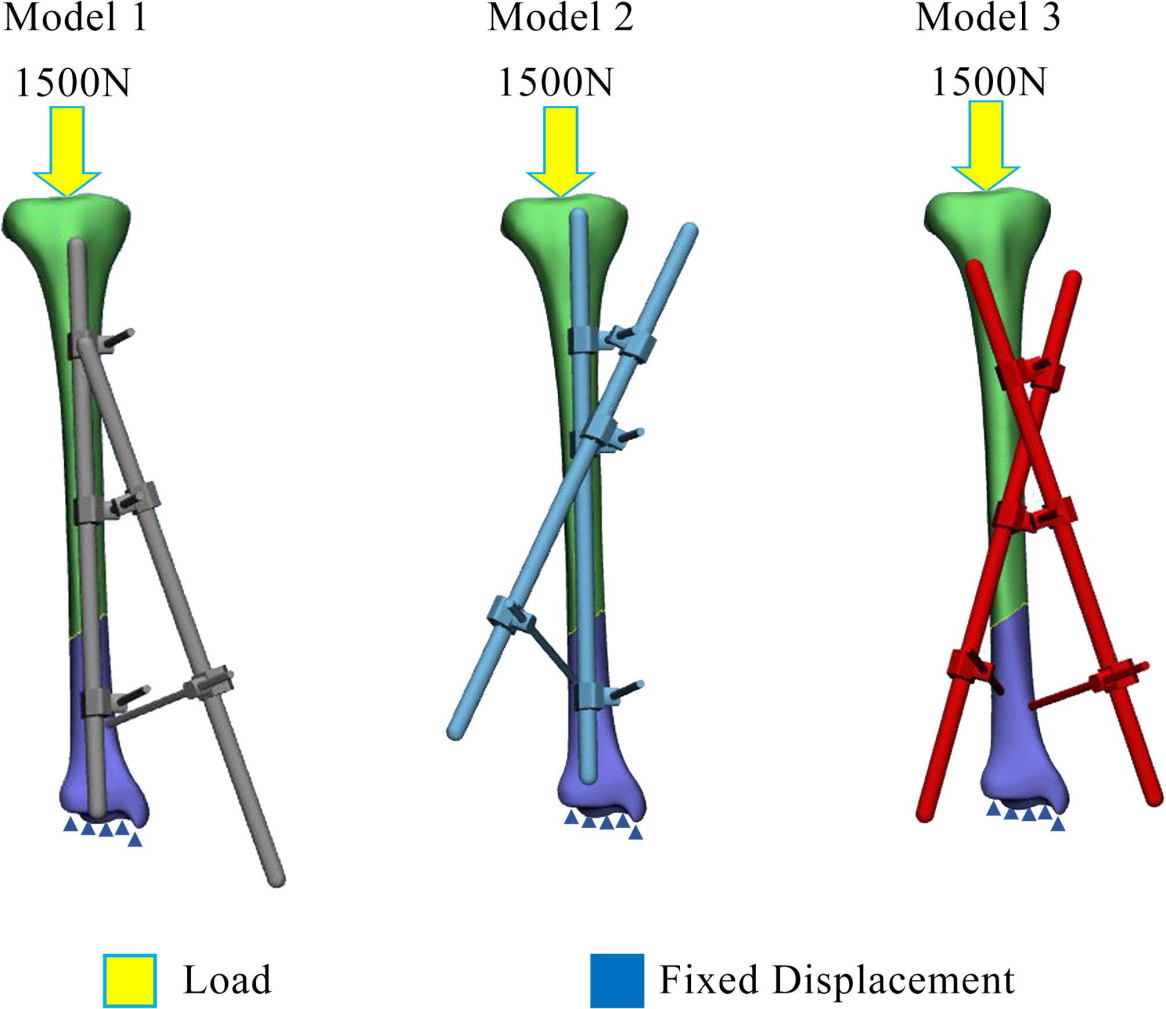
Boundary condition for analyzing three configurations of external fixator in treating oblique fracture.

Patient A, a 59-years-old lady, presented to the hospital after a motor vehicle accident with pain over the right leg and right ankle. Clinical examinations revealed a 5 cm x 6 cm laceration wound at the anterior aspect of her right shin with protruding bone, deformity over her right leg, and swelling over her right knee. A diagnosis of open fracture of distal third right tibia was made. Other diagnosis such as closed fracture supracondylar right femur, closed fracture right medial malleolus, and closed fracture right fibula were made with the help of radiographs. There was no other injury upon completion of the secondary survey. Intravenous (IV) Cefuroxime 1 g stat was given upon admission. After stabilisation of her clinical conditions, the patient underwent wound debridement and exploration of right tibia wound, external fixation of distal third right tibia (Figs. 1 and 2), open reduction and distal femur locking plate insertion of the right femur, screw fixation of right medial malleolus, and intramedullary K-wire insertion of right fibula. In this case, the construct of Model 3 (Fig. 3) was utilised. No complication was noted during the post-operative period. Physiotherapy and quadriceps strengthening exercises were prescribed in ward. Range of motion of the knee prior to discharge was 0° to 90° flexion. Patient was discharged with non-weight bearing crutches and given clinic follow-up.

### Diagnosis and treatment for patient B

Patient B, a 20-years-old girl, presented to the hospital after being hit by a car while she was crossing the road. Upon examination, there was a wound over the lateral aspect of her right shin measuring 4 cm x 2 cm with active bleeding and deformity over her right tibia. Radiograph of the right leg showed comminuted fracture of distal third of right tibia with bone loss. Patient was transported to the operating theatre for wound debridement, exploration, suturing of right tibia wound, and external fixation and K-wire of right tibia, where Model 2 (Fig. 3) was utilised. Post-operative period was uneventful. Patient was treated with intravenous antibiotics and analgesics. Range of motion exercises were prescribed and patient was able to obtain 0^0^ to 90^0^ flexion right knee range upon discharge. Patient was discharged well with non-weight bearing crutches and was given clinic follow-up.

### Three-dimensional modelling

Computer-aided design (CAD) software, Mimics (Materialise, Leuven, Belgium) was utilised to reconstruct a three-dimensional (3D) model of the tibia cortical and cancellous bones from computed tomography (CT) images. The CT scan process on a healthy person was ethically approved by ethical committees from Hospital Tengku Ampuan Afzan Kuantan, under Ministry of Health Malaysia. The thickness of axial slices of the CT images was 1.5 mm. Both cortical and cancellous bones were differentiated using different Hounsfield Unit (HU) values, in which the HU values represented the bone density threshold value. The HU values higher than 700 was set for cortical while values lower than 700 was for cancellous bone. To make sure the model is reliable to be used, a validation work was conducted and reported in the previous published literature^6^, in which five different locations of strain were measured in the experimental work and compared with the predictions results from finite element model. From the validated model, an oblique fracture was created on the 3D constructed tibia. For the external fixator construct, three different configurations were designed in another CAD software, Solidworks (Dassault Systèmes SolidWorks Corp., USA). The rods diameter was set to 11 mm and pins diameter set to 5 mm. All three external fixators models were then saved into stereolithography (STL) format. To complete the configurations of 3D model of bone external fixator, all STL files were imported into 3-matic software (Materialise, Leuven, Belgium). Model 1 consists of one rod placed in-line to the tibia, while a second rod was set in oblique position on one of the distal and proximal pins. This construct can be known as a non-cross external fixator configuration. Model 2 was constructed similar to Model 1 in which one rod was in-line to the tibia bone, but with the oblique rod fixed to both proximal pins. This construct can be referred as a single cross external fixator configuration. Model 3 is the novel configuration by the authors where two oblique rods were fixed on both proximal pins on either side of the rods while the distal end is fixed with pins to the distal fragment of the bone. This can also be referred as double cross external fixator configuration. Figure 3 illustrates all three constructs in this study. To be noted, the configuration of the external fixator in Model 3 (Fig. 3) was similar to that of patient’s fixator as shown in Figs. 1 and 2.

### Finite element analysis

From the 3-matic software, all models were meshed with first order tetrahedral elements and saved in volume files. The optimum tetrahedral mesh size for bone and external fixator was obtained through a previous convergence study^19^. The mesh size of the bone was set as 3 mm and external fixator was at 1 mm^19^. Later, the volume files were then imported into Marc Mentat software (MSC Software, Santa Ana, CA) for finite element analysis. All models were assigned with linear isotropic and homogeneous material properties. Young’s modulus (E) of 16,000 MPa^18^ and 1,100 MPa^19^ were set at the cortical and cancellous bone, respectively. For Poisson’s ratio (v), it was set at 0.26 and 0.3 for the cancellous and cortical bone, respectively. As for the external fixator, it was assigned with the titanium material properties, in which the E and v were set at 110,000 MPa and 0.3, respectively^19^. Meanwhile, a contact between the cancellous and cortical bone was set as glue condition in which fully bonded with no friction coefficient. The contact between external fixator and bone, as well as for the fracture fragments, were set as a touch condition (non-bonded) at the friction coefficient of 0.3^13^. In terms of the boundary condition, the region of distal tibia bone was fixed in all degree of freedom, meanwhile an axial load of 1500 N was applied to the region of proximal tibia bone in order to simulate axial compression^13^.

## Results

### Clinical and radiography outcomes

Patient A was comfortable and did not experience any pain throughout her tibia external fixator usage period. No pin site infection and loss of reduction were found in serial radiographs taken during follow-up despite allowing the patient to partial weight bear. However, the condition of patient was complicated with delayed union secondary to wound infection. The external fixation facilitated wound debridement and daily dressing where bone grafting and locking plate had to be done to stimulate bone growth after infection was eradicated. Patient resumed full weight bearing during the sixth month follow-up with 0 to 90° flexion knee range motion.

**Fig. 4:**
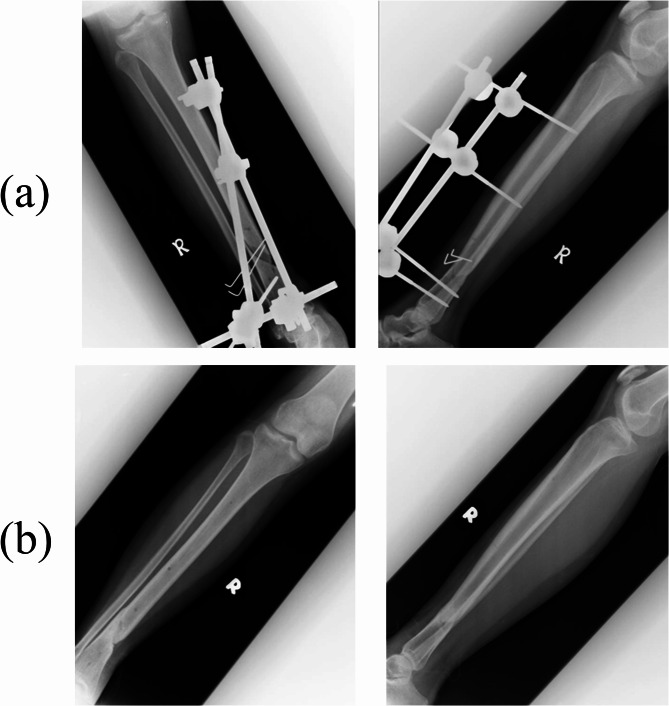
Radiographs of the single crossed locked rod in a comminuted distal 1/3 tibia fracture done on Patient B; (**a**) post-operative and (**b**) after heal.

Patient B was doing well during follow-up in which the external fixation was kept for a total of three months without any pin site complications. The patient did not experience any pain at rest throughout the healing process and was allowed with partial weight bearing during the second month of follow-up. During the third month follow up in the hospital, the external fixator was removed and patella tendon bearing cast was applied to allow full weight bearing. The patient was able to ambulate without cast on the fourth month follow-up (Fig. 4).

### Micromotion and stress distribution at fragment site

**Fig. 5:**
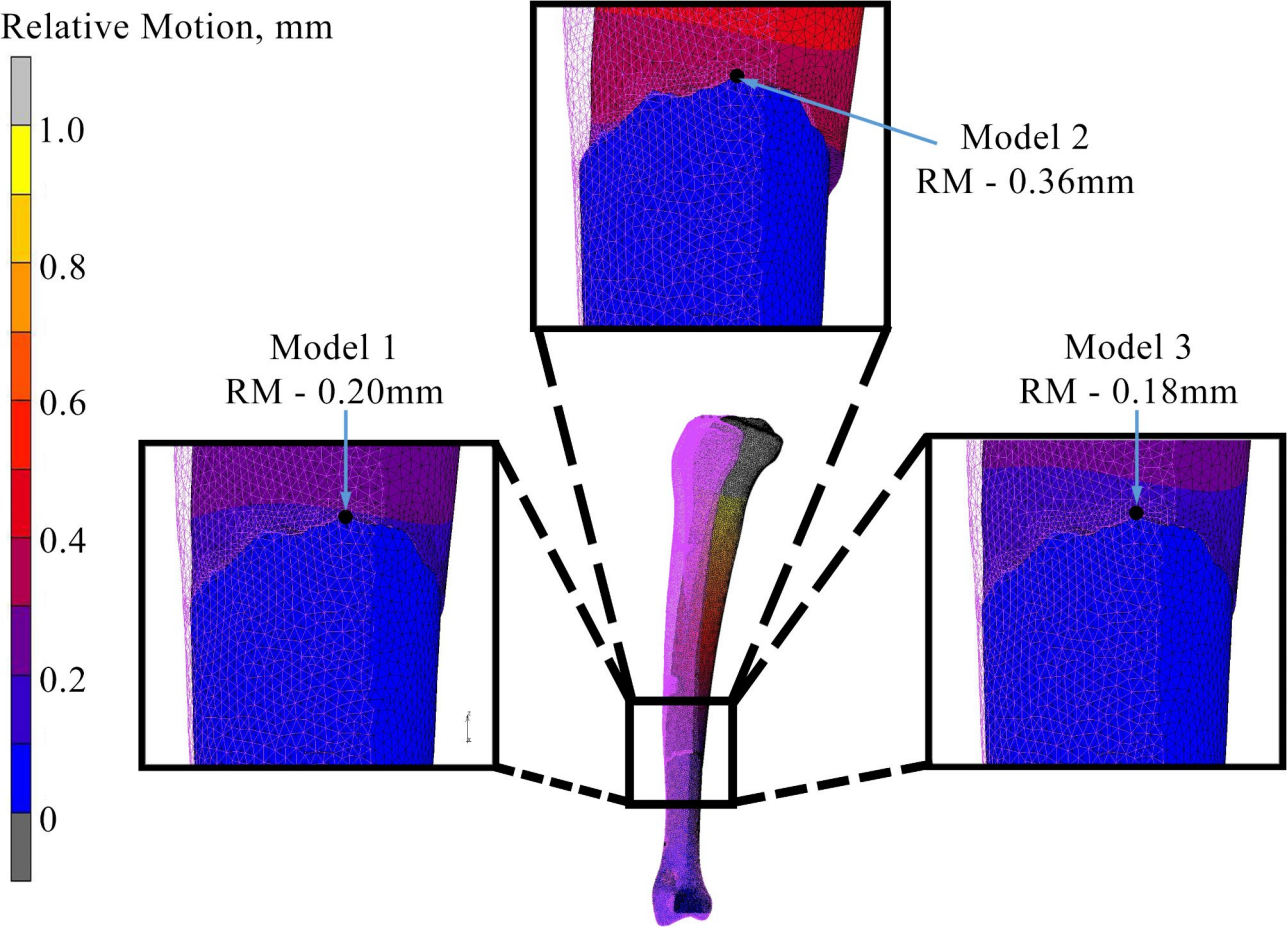
Relative micromotion at the fracture region between proximal and distal tibia.

**Fig. 6:**
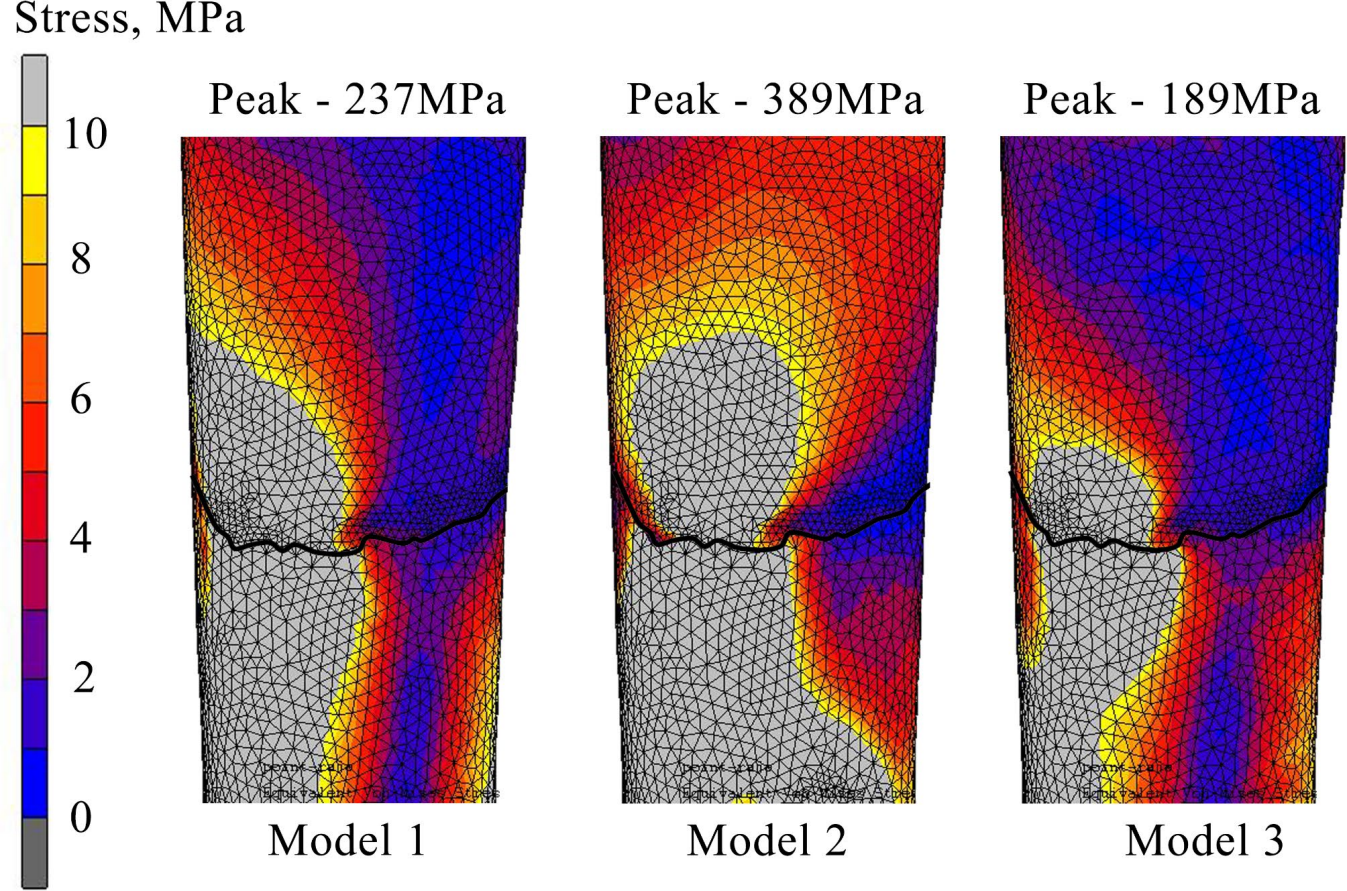
Stress distribution at the fracture region between proximal and distal. Thick line represent boundary for tibia fracture.

For the result of relative micromotion at the fracture fragment, FE analysis indicated that Model 2 (0.36 mm) had the highest micromotion and it was two-fold higher than Model 3 (0.18 mm), which had the lowest micromotion. While, Model 2 (0.20 mm) had 10% higher compared to Model 3. Figure 5 shows the relative micromotion for all constructs, where the wireframe and contour bones indicate the original position before and after load was applied, respectively. Meanwhile, the lowest peak stress value of 189 MPa was found at the model 3 (Fig. 6). Both Model 1 and Model 2 showed peak stress values of 237 MPa and 389 MPa respectively.

### Displacement and stress at external fixator

**Fig. 7:**
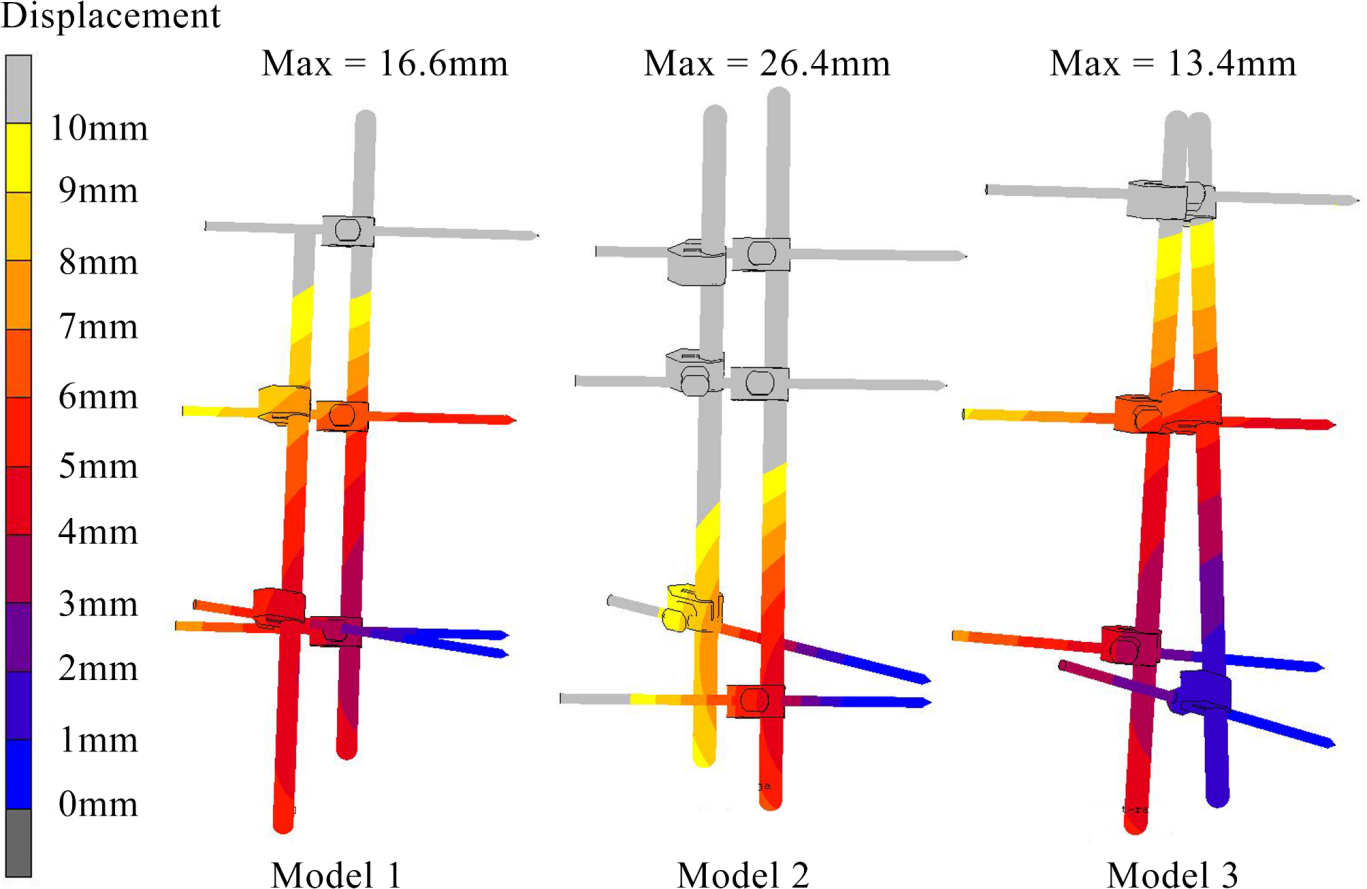
Displacement of three configuration of external fixator.

As for the displacement as shown in Fig. 7, the trend of it is similar with the relative motion at the fracture site (Fig. 5) where the model 2 demonstrated less stable as compared to model 1 and 3. The external fixator in Model 3 configuration was the most stable configuration, where displacement value was 13.4 mm. In contrast, the highest displacement was recorded by Model 2 configuration with 26.4 mm displaced. Meanwhile, Model 1 configuration had a displacement of 16.6 mm.

**Fig. 8:**
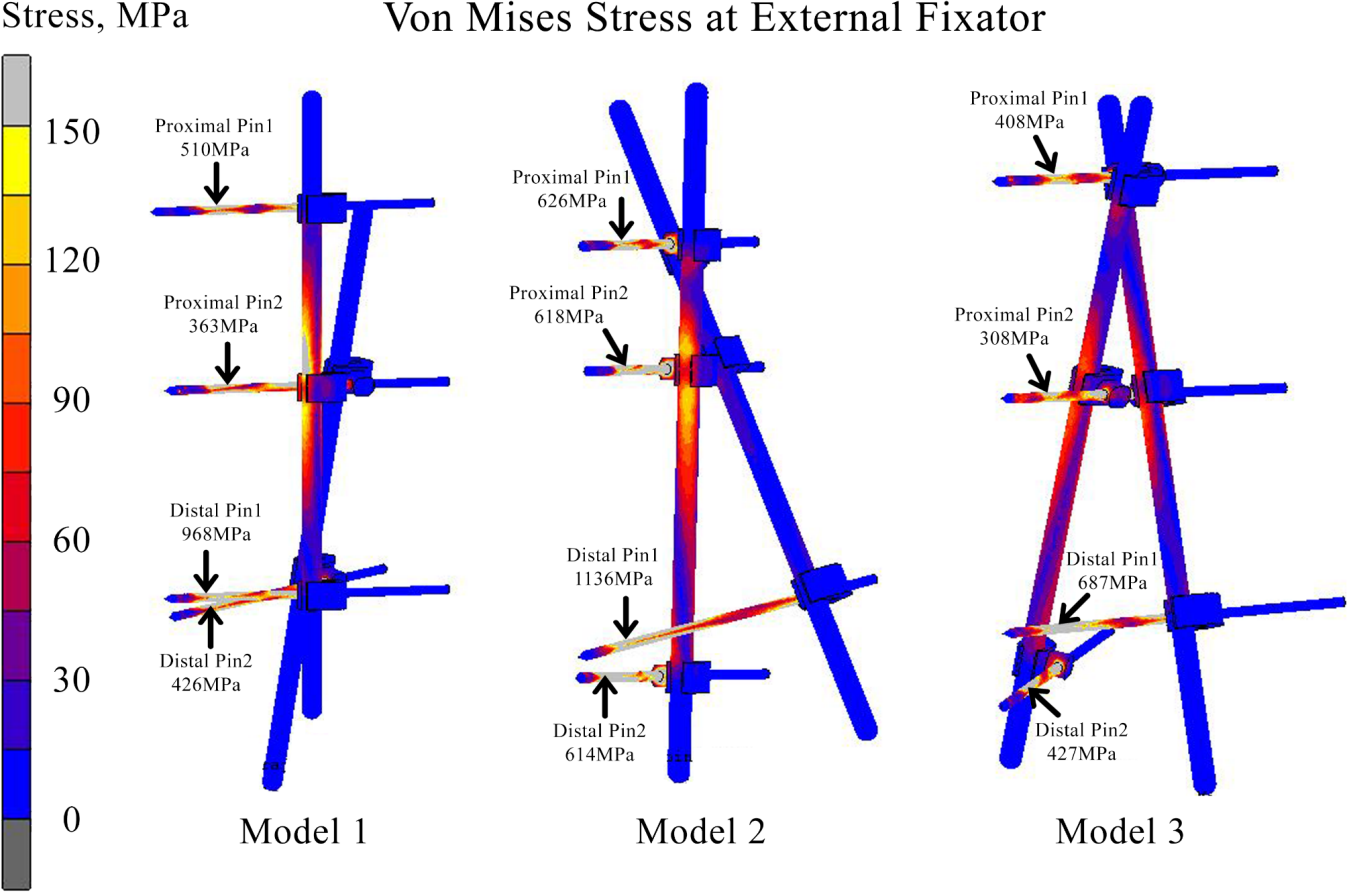
Stress distribution at 3 different configurations of external fixator.

Figure 8 shows the stress distribution and peak stresses at the pin region. From the figure, it is found that the model 3 produced the lesser peak von Mises stress at 1st, 2nd and third pin as compared with the Model 1 and 2. The 4th pin of the model 3 is slightly higher that model 1 with only at 1 MPa different. From other point of view, the maximum stress was found at distal pin 1 in all configurations. From the figure, the Model 2 recorded the highest maximum stress of 1136 MPa, while the maximum stress for Model 3 was 687 MPa. Meanwhile, the peak von Mises stress for Model 1 was demonstrated at 968 MPa which was 15% lower than Model 2. Findings from this study showed that the peak stress at the external fixator, particularly demonstrated at the pin-bone interface, in which near to the tip of each pin. Figure 8 summarizes the stress distribution at each pin of the external fixators for all three models.

## Discussion

External fixators can often be seen used in many clinical scenarios especially in cases caused by high energy trauma. Undoubtedly, external fixators could provide the ideal environment for both bone and soft tissue healing while at the same time permits additional procedures to be carried out to the site of injury without compromising reduction and alignment. At the same time, it also helps in providing a more comfortable condition for wound care. From a previous study by Patil et al.^2^, clinical outcomes showed good results in bone union of distal tibia fracture treated with delta frame external fixator as a definitive treatment device. The external fixator construct should not only provide optimal condition for fracture healing and enables easy access to wound or fracture site, but should also enable loading and partial weight bearing for better bone healing process^20^. This study was conducted to assess two clinical cases that utilized Model 2 and Model 3 (as shown in Fig. 3) and further FEA was done to biomechanically compare the stability and stress distribution of three different constructs of the external fixator when treating open distal tibia fractures.

From the findings, the construct built based on the cross-locking concept showed promise of providing a stable environment for bone healing process to take place. This was done through clinical observation and FEA. It is known that a too rigid fixation results in slower healing process^21^ while a less rigid fixation produces relatively high micromotion which could result in delayed union or even non-union fracture^21,22^. Previous experimental studies have reported that interfragmentary relative micromotion is an important element where the values between 0.15 mm and 0.4 mm have shown to assist healing of a fracture gap not more than 3 mm^23^. In 2009, two other studies obtained similar findings that stated fracture gap of below 3 mm had a threshold for relative micromotion of not more than 1.0 mm to avoid tissue damage and non-union^22,23^. The findings from this study shows that all configurations produced a result of relative micromotion that below than the threshold value. Therefore, all configurations in this study are not only able to provide a stable environment, but could possess minimal risk of disruption and other complications such as mal-union and misalignment during the bone healing conditions. Based on Fig. 5, the most stable configuration was Model 3, as the double cross-locking fixator demonstrated the smallest relative micromotion at 0.18 mm in FE analysis, followed by Model 1 with a value of 0.2 mm. To be noticed, Model 2 was found to be less stable since the FE analysis demonstrated the highest value of micromotion (0.36 mm). With this issue, it could lead to interruption in the formation of callus bone process^24^. Nevertheless, all configurations in the FE simulations had less than 1.0 mm relative micromotion at the fracture site which indicate optimal condition for bone healing is fulfilled^13^.

In the clinical and simulation observation, construct built based on the cross-locking concept was able to withstand intermittent loading. Generally, the healing process in fractured bones begin with proliferation of mesenchymal tissue at the fracture site and this leads to the formation of callus^25^. Intermittent mechanical stress plays an important role in mesenchymal cells differentiation^26^. To be noted, good blood supply coupled with low stress at the fracture fragment regions (Fig. 6), could possibly encourage bone healing with minimum condition of callus formation. In contrast, the high stresses at the fragment (Model 2 in Fig. 6) could prevent callus formation during the bone healing phases^25,27^. A study by Carter et al.^25^ where they mentioned that not only bone healing process can be delayed, but it contributes to fracture non-union condition if high compressive stresses occurred at the fracture region. This is also supported by other findings in 2012 and 2016, in which concentrated high stresses could possibly encourage long cells of connection issue development, and later lead to non-union complication^26,28^. The FEA results demonstrated that the von Mises stress distributed at the fracture region for Model 3 was the lowest as shown in Fig. 6. On the other side, the stress concentrated at the fracture region for Model 2 was twice higher than Model 3 and Model 1 was 20% higher than Model 3. Therefore, this finding could suggest that Model 3 is able to prevent complication of non-union at the fracture site.

This tested concept is safe to be employed without compromising pin-bone interface. The high stresses at the external fixators were associated with unstable fixation^19^. In several previous studies, the maximum stress was located at the interfaces of pin-bone where the magnitude of stress varied depending on the models and cases (300-800 MPa)^19,29^. Findings from this study agrees with the previous studies for all configurations (Fig. 8), where the peak stress was demonstrated at the pin-bone interfaces. Nevertheless, in terms of magnitude of stresses, the maximum stresses for all pins did not exceed the ultimate strength for Titanium (800-1110 MPa) which indicated that all configuration of external fixators are able to provide acceptable stability to avoid implant failure^30^. Several studies reported that improper pin placement could induce high rate of complications, where loosening and infections of pin demonstrated a maximum of 50% of clinical cases^31,32^. From the results, Model 2 has the largest displacement (Fig. 7) compared to others. As far as the biomechanics concept is concerned, this may lead to loosening at the pin-bone interface^29^.

The double cross-locking construct provided optimum rigidity while employing the same number of elements. Patients who sustained trauma to the distal tibia could benefit from the stability of this configuration without the additional weight^33^. This construct could provide better axial and rotational stability when compared to another hybrid fixator and delta frame construct. A lean construct without the added weight and space-occupying elements when comparing to other limb reconstruction system such as Orthofix, Ilizarov ring and Taylor spatial frame could enable easier post-operative rehabilitation, muscle strengthening exercises, encourage joint range of motion and at the same time ensure comfort. These properties are even more important in the already physically compromised patients. This simulation study had two cross-locking constructs, namely single cross-locking (Model 2) and double cross-locking (Model 3) and both utilizes the same number of elements. However, the double cross-locking external fixator was superior than others in terms of stress distribution, as shown in Figs. 7 and 8.

There are few limitations in this study which should be considered in future studies. External fixators with double cross-locking construct are a promising technique in terms of rigidity that can provide an optimum environment for bone healing process to take place. However, we also understand that reliance to finite element study with lack of biological modeling capabilits to assess the impact on bone healing is a significant limitation. Furthermore, timing component in bone healing is also essesntial and cannot be evaluated by finite element analysis alone. Furthermore, the results between clinical and simulation works were contradicted each other where Model 3 is for stiffer than Model 2 in the finite element analysis, while Model 2 allow patients to heal within 3 months as compared to Model 3 (6 months). To be noted, the bone healing in theses clinical cases could not be viewed as a standardized environment. The difference of time to union in both clinical cases are largely contributed to presence of other factors such as infections, demographic differences, extend and difference of energy in trauma. Thus, the difference in healing time should not be the main factor in evaluating this study’s concept. Moreover, this recent study has limited number of case studies and finite element analysis. Further study could be conducted by considering a number of different fracture configurations (spiral and transverse fracture) until the complex condition of fractures with bone loss. However, since the purpose of the finite element study was to compare three self-locking concepts of external fixator configurations in treating tibia fracture, therefore this study was focusing on a single fracture characteristic to ensure comparable results. Meanwhile, for the clinical assessment, it is only showing the justification and proof that the configuration could be considered to be used in traumatic cases in hospital. Nevertheless, more subjects should be involved in the future studies to ensure that the results are more reliable and provide strong evidence of the safety and healing process. Another limitation from this study is that the other parameters in constructing the external fixator configurations were not considered. Those parameters including impact of pin size and location, bar size, tightening torque of the clamp, impact of soft tissue envelope on stability, callus volume over time, and pin coating or insertion technique. Therefore, it is recommended that future studies on these parameters could be done by our team and other researchers.

For the computational simulation, several limitations and assumptions were made throughout the study. Firstly, the tibia bone was reconstructed from CT image data of healthy subjects with a virtually constructed fracture. This may affect the predictions and outcome albeit being an acceptable methodology commonly used by other researchers^34,35^. CT image data of real patients should be used in the FEA in future studies. Secondly, the use of isotropic and homogenous properties for the cortical and cancellous bones may also alter the predictions. It should be noted that the real bone has inhomogeneous material properties. However, current computer resources are not able to perform simulation of inhomogeneous model and the method described here has also been used by many other scholars^34,35^. Moreover, this study is only focus on one fracture which is distal third tibia fracture. Thus, other finite element simulations could be done to study the effect of different fracture patterns and more complex on fracture model such as pilon fracture. In this simulation, there was only one load condition which is axial force. This is another limitation of study since it is only a pre-liminary investigation on the novel configuration. To increase the confidence and produce more reliable results, it is suggested to include more load conditions such as bending and torsion in the future. Moreover, the finite elemenet analysis in this study was focused on a single static load boundary condition, therefore it is suggested to include cyclic biomechanical testing in the nearest future studies where this could perhaps analyse many aspects of mechanical stability in terms of pin and clamps loosening, and overall stiffness.

## Conclusions

As a conclusion, the authors believe that this cross-locking concept could be employed easily by using elements widely available in any hospitals. This is to increase the stability of the construct used in the treatment of distal third tibia fracture without the need to increase the number of elements or size of pins and rods used, as evident by this clinical observation and FEA study. Based on the outcomes from finite element analysis, it is found that Model 3 construct is the most stable configuration and should be viewed as an option for medical surgeons to treat patients, where the Model 3 illustrated successful outcomes in clinical study for Patient A and comparable with Model 2 for Patient B. These outcomes show that both models are acceptable in the clinical practises with promising healing process.

**Statement of originality**.

The authors declare that this manuscript is original, has not been published before and is not currently being considered for publication elsewhere. The authors confirm that the manuscript has been read and approved by all named authors and that there are no other persons who satisfied the criteria for authorship but are not listed. The authors further confirm that the order of authors listed in the manuscript has been approved by all of us. The authors understand that the Corresponding Author is the sole contact for the Editorial process. He/she is responsible for communicating with the other authors about progress, submissions of revisions and final approval of proofs.

No additional information is available for this paper.

## Data Availability

The necessary data used in the manuscript are already present in the manuscript.

## Declarations

### Declaration of AI use

The authors declare not use AI-assisted technologies in creating this article.

### Transparency statement

The authors affirm that this manuscript is an honest, accurate, and transparent account of the study being reported; that no important aspects of the study have been omitted; and that any discrepancies from the study as planned (and, if relevant, registered) have been explained.
